# Mitochondria-Targeted Antioxidant SS-31 is a Potential Novel Ophthalmic Medication for Neuroprotection in Glaucoma 

**Published:** 2015

**Authors:** Yu PANG, Chao WANG, Ling YU

**Affiliations:** Department of Ophthalmology, the Affiliated Hospital of Luzhou Medical College, Luzhou, Sichuan Province, China.

**Keywords:** Glaucoma, Mitochondria-targeted antioxidant SS-31, Neuroprotection, oxidative stress mitochondrial dysfunction

## Abstract

Glaucoma is the second leading cause of irreversible blindness and a neurodegenerative disease with a complex pathogenesis. Increasing evidence suggests that oxidative stress and mitochondrial dysfunction have crucial roles in most neurodegenerative diseases such as glaucoma. The conventional clinical treatment for glaucoma is lowering the intraocular pressure (IOP). Some patients have normal IOP, whereas other patients appear to obtain adequate control of IOP after filtration surgery or medication. However, these patients still experience progressive visual field loss. Vision field loss in glaucoma is attributed to retinal ganglion cell (RGC) apoptosis. Many recent researches demonstrated that the link between mitochondrial dysfunction and oxidative stress was a major cause of RGCs apoptosis. How oxidative stress leads to RGCs apoptosis in glaucoma is unclear but may involve the neurotoxic effects of oxidative stress-induced mitochondrial dysfunction and/or damage from reactive oxygen species (ROS). Investigations are needed concerning the mitochondria as effective targets for potential therapeutic interventions to maintain mitochondrial function and reduce oxidative stress, and thereby delay or stop RGC loss and prolong visual function. The mitochondria-targeted antioxidant Szeto-Schiller (SS) peptide is a candidate molecule. Szeto-Schiller-31 (H-D-Arg-Dmt-Lys-Phe-NH2) is an attractive mitochondria-targeted antioxidant that can protect the mitochondria and RGCs against oxidative damage. Therefore, we suggest SS-31 as a novel neuroprotective ophthalmic drug for protecting RGCs in glaucoma.

## INTRODUCTION

Glaucoma is increasingly recognized as a chronic neurodegenerative disorder characterized by optic nerve head cupping and visual field defects caused by damage to and apoptosis of RGCs. Glaucoma treatment focuses on lowering IOP via pharmacotherapy or surgery. Epidemiological investigators have recently reported that lowering IOP alone cannot prevent the progressive loss of visual field in glaucoma patients. There is improved knowledge about the mechanism of optic nerve damage. As a consequence, the concept of neuroprotection has been increasingly proposed. Additional neuroprotective strategies may offer novel treatments to prevent RGCs loss and disease progression. Increasing IOP is a major risk factor in glaucoma; however, other concomitant factors also significantly affect the eye such as oxidative stress caused by ROS [1], increased glutamate levels [2], toxic effects and vascular alterations [3]. Recent accumulating evidence suggests the involvement of mitochondrial dysfunction in glaucoma [4,5]. Oxidative stress is a common manifestation of mitochondrial dysfunction, and it has been repeatedly implicated in the pathogenesis of glaucoma [6].

**Oxidative stress and mitoc** **hondrial dysfunction in glaucoma**

In brief, oxidative stress is an imbalance between processes that generate ROS and processes that remove them. More than two decades ago, oxidative stress was first proposed as a contributor of glaucoma pathogenesis [7]. Many clinical and experimental studies have assessed ROS production, antioxidant levels, and macromolecules involved in oxidative damage under glaucomatous stress. For example, in an experimental rat model of glaucoma, intracameral injection of hyaluronic acid decreased antioxidants and increased lipid peroxidation in the retina [1]. In the experimental glaucoma models, cauterization of the episcleral veins in rat leads to ocular hypertension, and the levels of ROS, nitrite as well as lipid peroxidation are markedly increased [8,9]. In another study, the researchers injected the hypertonic saline into the episcleral vein to get the increased IOP. Also, protein oxidation was detected localizating in the inner retinal layers including RGCs [10].

The mitochondria are normally protected from oxidative damage, owing to mitochondrial antioxidant systems, a multilayer network; however, if ROS production exceeds the antioxidant capacity of mitochondria, these cells experience oxidative damage [11]. With the advent of oxidative stress, excessive ROS act as signaling molecules to activate apoptotic pathways. ROS can react with neighboring molecules in the mitochondria such as nucleic acids, proteins, and lipids, and subsequently induce cell death [12]. The mitochondria perform many tasks to maintain biochemical events. Mitochondrial function, especially the generation of adenosine triphosphate (ATP), is necessary for neuronal survival. All neuronal degeneration is associated with mitochondrial dysfunction [13,14]. During oxidative damage, the mitochondria are impaired and generate ROS more frequently than ATP. Oxidative stress and ATP depletion lead to RGCs mitochondrial dysfunction, and result in RGCs death. Oxidative damage to mitochondria leads to mitochondrial permeability transition (MPT), mitochondrial depolarization, mitochondrial swelling, further excessive ROS production and the cell death mediators such as cytochrome c (cyt c) and apoptosis-inducing factor (AIF) releasing [15].

A pathological feature of glaucoma is apoptosis of RGCs. Caspase mediation activates the proteolysis cascade, which can trigger the apoptosis of RGCs by different stimuli [16,17]. Caspases disrupt the electron transport chain of mitochondria resulting in mitochondrial dysfunction and the generation of ROS [18]. McKinnon [19] states that caspase-3 and caspase-8 were involved in RGCs apoptosis in experimental rat models of glaucoma. In experimental rat models of glaucoma, it is demonstrated that mitochondrial dysfunction has been related to RGCs apoptosis [20,21]. In vitro studies show that caspase-8 activation can occur downstream of mitochondrial dysfunction [22,23]. Caspase-8, which cleaves and activates all other caspases, has an important role in triggering apoptosis by activation of the caspase cascade. In glaucoma, the transcription factor p53 activates the proapoptotic Bax protein, which leads to RGCs apoptosis via a caspase-3–dependent pathway [24]. This process is required for cyt c release from the mitochondria [25]. Normally, Cyt c is bound to the inner mitochondrial membrane (IMM) in association with cardiolipin [26]. With MPT, calcium overload [27] or peroxidation of cardiolipin [26], cyt c is released from the IMM to the cytosol via the opening of MPT pores [28]. In the cytosol, cyt c combines with Apaf-1 (a cytosolic factor) to form an apoptosome, which then binds procaspase-9 [29]. The active caspase-9 cleaves caspase-3 and caspase-7, which then continue to cleave specific substrates within the cell [28, 30]. Caspase-3 is a common downstream caspase that mediates RGCs apoptosis. Caspase activation induces ROS production and loss of the mitochondrial transmembrane potential, which provide feedback to the insult to mitochondrial function. Mitochondrial dysfunction is thereby aggravated. By contrast, ROS initiate the death pathway by poly (ADP-ribose)polymerase-1-mediated cleavage of AIF [31]. Apoptosis-inducing factor translocates from the mitochondria to the nucleus. It can then directly induce RGCs death via chromatin condensation, DNA fragmentation and nuclear shrinkage [32]. Tezel et al demonstrated that reducing ROS generation could temporarily protect RGCs from apoptosis [33]. Oxidative stress, increased ROS production, and mitochondrial dysfunction have emerged as an oxidative stress-mediated mitochondrial vicious cycle that promotes RGCs loss.

Based on the aforementioned evidence, we can understand that mitochondrial dysfunction in combination with oxidative stress orchestrates the apoptosis of RGCs by activating different stimuli. Mitochondrial dysfunction is responsible for cytotoxic events in RGCs apoptosis. Enhancing mitochondrial function could prolong RGCs survival. Oxidative damage to mitochondria leads to hyperpolarization of mitochondrial membrane potential, which causes mitochondrial dysfunction and releases stimulative apoptotic compounds. In addition, mitochondria are major generators of ROS and scavenge ROS to reduce ROS-evoked apoptosis signaling. We suggest that a targeted mitochondrial antioxidant could be a viable therapeutic intervention.

## HYPOTHESIS

Retinal ganglion cells require high energy. They rely on the mitochondria for survival and function. Pre-existing congenital or acquired mitochondrial dysfunction can increase the vulnerability of RGCs to stress from risk factors [34]. Alterations in the functional status of RGCs axon mitochondria will influence RGCs survival in glaucoma [35]. A pharmacological agent that effectively ameliorates oxidative stress and enhances RGCs’ mitochondrial function should be a new treatment approach to reduce the rate of visual field loss in glaucoma. A variety of publications have suggested the use of antioxidant supplementation to help in the treatment of glaucoma [36,37]. Among the many methods, we have concentrated on the mitochondria-targeted antioxidant SS-31 and hypothesize that SS-31 may be a logical therapeutic intervention for neuroprotection in glaucoma.

**Evaluation of the hypothesis**

**Figure 1:**
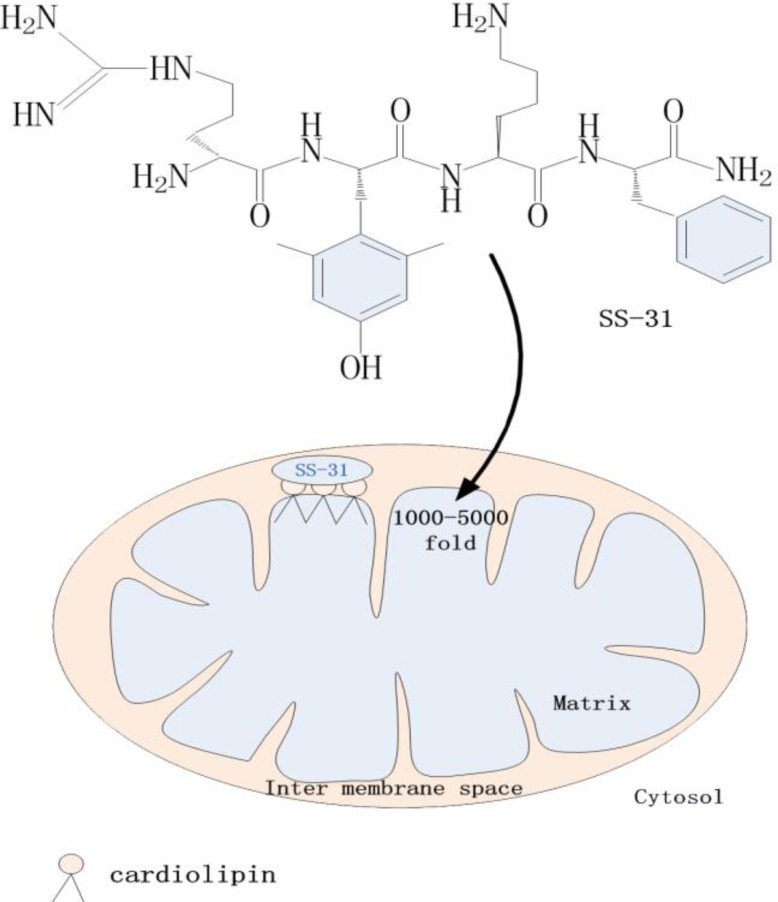
Schematic representation of a mitochondrion taking up SS-31. This molecule selectively binds to cardiolipin and accumulates 1000- to 5000-fold in mitochondria. Mitochondrial uptake of SS-31 does not depend on the mitochondrial transmembrane electric potential.

Hazel Szeto and Peter Schiller discovered a group of small synthetic peptides, called SS peptides, which are limited to fewer than 10 amino acid residues and contain alternating aromatic residues and basic amino acids. There are three peptide analogues in this series: SS-02, SS-31 and SS-20. Each molecule carries a 3+ net charge at physiological pH [38]. These peptides are solid-phase [39], stably water soluble, and designed to resist peptidase degradation [40]. Zhao found that these peptide antioxidants are cell-permeable and are concentrated by 1000-fold in the IMM. They are taken up into the IMM in an energy-independent, non-saturable manner [15]. This is clearly an advantage (Figure 1).

Compared to general antioxidants such as vitamin E, brimonidine, and other biological antioxidants, mitochondrial-targeted peptides show more potential therapeutic neuroprotective effects in some research. General antioxidants tend to be poorly cell-permeable and quite susceptible to the mitochondria. They have an unsatisfactory curative effect because they do not reach the relevant sites of free radical generation within the mitochondria. Vitamin E and coenzyme Q are mitochondrial-targeted antioxidants, and they are very lipophilic. They are taken up into mitochondria by exploiting the potential gradient across the IMM [41]. However, they require high concentrations to cause mitochondrial depolarization [42,43]. Therefore, we believe that mitochondrial-targeted peptides may provide greater neuroprotective effects.

Increasing evidence supports SS-31 as superior to the other SS peptides analogues such as SS-02 and SS-20. A few studies have shown that SS-31 can scavenge mitochondrial ROS in a dose-dependent manner because it possesses a tyrosine residue [15]. However, because it lacks a tyrosine residue, SS-20 cannot scavenge ROS in the same concentrations [15]. Mitochondrial ROS contribute to MPT and mitochondrial swelling; therefore, by reducing mitochondrial ROS, two scavenging SS peptides (i.e., SS-02 and SS-31) were able to inhibit MPT, prevent mitochondrial swelling, reduce cyt c release, and further reduce oxidative damage of mitochondria [44]. The peptide SS-02 was confirmed by uptake studies with [3H]SS-02 present in isolated mouse liver mitochondria at a 1000-fold concentration [15]. Using the same methods, Zhao et al showed that [3H]SS-31 was also rapidly taken up into isolated liver mitochondria, and its levels were nearly five-fold higher than those of SS-02 [45]. Based on these studies, we concluded that SS-31 is the most efficient peptide in this series.

**Figure 2:**
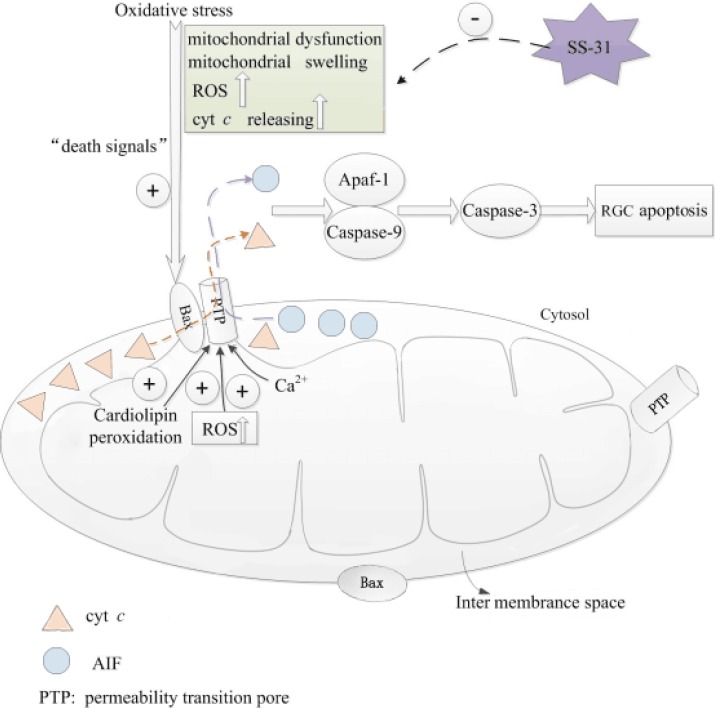
Simplified scheme of oxidative stress-induced mitochondrial pathway of apoptosis. Oxidative stress leads to mitochondrial dysfunction, reactive oxygen species (ROS) production, mitochondrial swelling, and the release of cytochrome c (cyt c), and apoptosis-inducing factor (AIF) from the intermembrane compartment to the cytosol. These actions are “death signals.” The ROS production, calcium overload or peroxidation of cardiolipin promote permeability transition pore (PTP) opening. Once in the cytosol, cyt c and AIF, in cooperation with Apaf-1, activate caspase-9 and other members of the caspase family, trigger apoptosis, and eventually lead to retinal ganglion cell (RGC) apoptosis. The peptide SS-31 can reduce mitochondrial ROS production, inhibit cyt c release, and prevent mitochondrial swelling in isolated mitochondria. Activation is indicated by the symbol⊕ and inhibition by the symbol ϴ.

The peptide SS-31 can inhibit MPT, reduce mitochondrial ROS production, and prevent mitochondrial swelling in isolated mitochondria [15]. In addition, SS-31 can scavenge ROS and inhibit lipid peroxidation in vitro [45]. As a mitochondrial-targeted peptide, SS-31 has been evaluated in some neurodegenerative disease models such as in islet cell transplantation [46], myocardial infarction [47], brain ischemia reperfusion [48], amyotrophic lateral sclerosis [49] and Alzheimer’s disease [50]. In all of these conditions, SS-31 exhibited remarkable neuroprotective effects (Figure 2).

In the field of ophthalmology, Huang and colleagues [51] assessed the protective action of SS-31 in the retinas of diabetic rats. They reported that the most prominent neuronal abnormality in diabetic rats is apoptosis of RGCs [52] and that the RGCs of diabetic rats express several proapoptosis molecules that are very vulnerable to oxidative stress [53]. The authors observed that SS-31 could protect retinal structures and inhibit the breakdown of the inner blood retinal barrier (iBRB) by reducing oxidative damage and thus preventing apoptosis of RGCs [51]. The mechanism of the results indicated that iBRB dysfunction was detected by the presence of extracellular fluid in the retina and sequential RGCs exposure to serum constituents, and consequently result in RGC apoptosis, which could result in immunoreactions with immunoglobulins and neurotoxicity with increasing glutamate levels [54]. In their study, they also found that SS-31 treatment promoted antiapoptotic protein expression and inhibited apoptosis-related protein overexpression in the retinas of diabetic rats [51]. We suggest that SS-31 may reduce cell apoptosis and prevent mitochondrial dysfunction, thus restoring retinal function so that RGCs are protected from apoptosis. In addition, a previous study demonstrated that SS-31 could decrease ROS production and prevent the release of cyt c from the mitochondria, which attenuates high glucose-induced injuries in human retinal endothelial cells [55]. Moreover, neuroprotection in glaucoma requires preventing RGCs apoptosis induced by oxidative damage. The opinions of Huang et al sustained our hypothesis that SS-31 could be a potent ophthalmic drug to prevent the apoptosis of RGCs and protect the optic nerve in glaucoma.

## CONCLUSION

Glaucoma is a multifactorial optic neuropathy characterized by progressive degeneration of RGCs. Oxidative stress occurs in glaucoma and contributes to RGCs degeneration. Mitochondria have a central role as generators and as targets of oxidative stress, and there has been growing evidence of mitochondrial dysfunction in the pathogenesis of glaucoma [56]. We suggest that the targeted mitochondrial antioxidant SS-31 has therapeutic potential to ameliorate the mitochondrial vicious cycle and thus protect RGCs from glaucomatous degeneration. To assess whether SS-31 could be a potent neuroprotective drug in glaucoma, further studies on oxidative damage in experimental glaucoma and clinical trials evaluating SS-31 treatment should be performed.
